# Ultrasound-guided small-bore chest drain placement: a retrospective analysis of feasibility, safety and clinical implications in internal medicine ward

**DOI:** 10.1007/s40477-025-01000-3

**Published:** 2025-02-28

**Authors:** Andrea Boccatonda, Viola Tallarico, Stefano Venerato, Carla Serra, Susanna Vicari

**Affiliations:** 1 Internal Medicine, Bentivoglio Hospital, AUSL Bologna, 40010 Bologna, Italy; 2 Department of Medical and Surgical Sciences, University of Bologna, 40138 Bologna, Italy; 3 Diagnostic and Therapeutic Interventional Ultrasound Unit, IRCCS Azienda Ospedaliero-Universitaria Di Bologna, 40138 Bologna, Italy

**Keywords:** Pleural effusion, Ultrasound, Chest drain, Internal medicine, Lung

## Abstract

**Purpose:**

Massive and complex pleural effusions represent a frequent challenge for internists, particularly when patients present with significant symptoms and the hospital setting lacks dedicated thoracic surgery or interventional pneumology services.

**Methods:**

This retrospective study evaluates the effectiveness and feasibility of ultrasound-guided small-bore chest drain placement performed by internal medicine physicians with interventional ultrasound experience. We analyze procedural success rates, complication profiles, and subsequent clinical management in a cohort of patients managed in a single internal medicine ultrasound ward.

**Results:**

In our series of ten patients, ultrasound-guided drain placement was successful in all cases. No immediate major complications were encountered, and subsequent complications were minimal and manageable.

**Conclusion:**

Ultrasound-guided small-bore chest drain placement is a feasible, safe, and effective alternative to surgical chest tube insertion in selected patients in internal medicine wards, potentially avoiding the need for hospitalization or transfer to specialized thoracic surgery services.

## Introduction

The management of massive and complex pleural effusions is a common challenging scenario for internists [1, 2]. These effusions may arise from various etiologies including parapneumonic processes, empyema, malignant involvement, and hydro-pneumothorax in patients with underlying congenital abnormalities [1, 2]. Pleural effusions can be categorized based on their etiology, size, and complexity [2, 3]. Massive effusions and complex, loculated collections can lead to significant respiratory distress, hypoxemia, and discomfort. In the oncology population, pleural effusions often serve as a marker of advanced disease and are associated with a limited prognosis, yet their management is crucial for palliative care and symptom relief [1]. Similarly, patients with infectious or inflammatory pleural diseases such as empyema require prompt and effective drainage to prevent progression to sepsis and multiorgan failure [4].

Traditionally, the management of those conditions involved the placement of chest drains by thoracic surgeons or interventional pulmonologists [5, 6]. However, many institutions, especially those without dedicated thoracic surgery or interventional pneumology departments, must rely on the expertise of internal medicine/emergency physicians. Therefore, internists have increasingly assumed responsibility for performing a range of interventional procedures, including thoracentesis and chest drain placement. This shift not only optimizes resource allocation but also improves the continuity of care within the medical department. However, the expansion of procedural responsibilities among internists necessitates rigorous training in interventional ultrasound techniques to ensure both efficacy and safety.

Over the past decade, the incorporation of ultrasound guidance into interventional procedures has markedly improved procedural safety and efficacy, particularly in the context of pleural interventions. Ultrasound guidance offers several advantages in chest drain placement. It provides real-time visualization of the pleural space and adjacent structures, reducing the risk of iatrogenic injury such as lung puncture, intercostal vessel laceration, or inadvertent injury to intra-abdominal organs. Moreover, it facilitates the selection of the optimal site for drain insertion, particularly in patients with loculated or complex effusions. The present study focuses on the use of small-bore chest drains (10.2 French) placed using the Seldinger technique, which is less invasive compared to traditional large-bore chest tubes and has been associated with lower complication rates and improved patient comfort [7–10].

## Study aims

The primary aim of this study was to evaluate the feasibility and effectiveness of ultrasound-guided small-bore chest drain placement performed by internal medicine physicians in a hospital setting without thoracic surgery or interventional pneumology support. Our secondary aim was to assess the complication rates—both immediate and delayed—and to determine the clinical implications of this approach, including its impact on patient management and disposition.

## Materials and methods

### Study design

We conducted a retrospective descriptive analysis of all cases in which small-bore chest drains were placed under ultrasound guidance in 2023 within the internal medicine ward at Bentivoglio Hospital (AUSL Bologna, Italy). This study was approved by the institutional review board (CE-AVEC 0013121). This study was performed according to Good Clinical Practice regulations (Good Clinical Practice for Trial on Medicinal Product—CPMP/European Commission-July 1990; Decreto Ministeriale 27.4.1992—Ministero della Sanità) and the Declaration of Helsinki (Hong Kong 1989). By signing the protocol consent form, study participants committed themselves to adhere to local legal requirements.

### Data collection

Data were collected on patient demographics, comorbidities, indications for drain placement, procedural details, immediate complications (such as intercostal artery injury, abdominal organ injury, iatrogenic pneumothorax, and subcutaneous hematoma), and complications observed at 24 h post-procedure. Additional data regarding the need for subsequent interventions (e.g., thoracoscopy) and patient disposition (discharge home, transfer to another ward, hospice) were recorded.

## Statistical analysis

Demographic, clinical, and outcome variables were described. Categorical variables were described as proportions and continuous variables as medians with their respective interquartile range (IQR).

## Results

### Patient demographics and clinical profile

The study cohort consisted of ten patients with a mean age of 71 ± 12.4 years. Among comorbid conditions, atrial fibrillation was noted in 60% of patients, type 2 diabetes mellitus in 60%, and active neoplasia in 40%. Notably, anticoagulation therapy was present in 60% of the cohort, increasing the potential risk for bleeding complications during invasive procedures. Patient’s characteristics were summed in Tables 1 and 2.

**Table 1:** Demographic and clinical characteristics of the patient cohort

Characteristic	Number of patients (%)
Male sex	8 (80%)
Comorbidities	
COPD	2 (20%)
Atrial fibrillation	6 (60%)
Hypertension	4 (40%)
Type 2 diabetes mellitus	6 (60%)
Active neoplasia	4 (40%) (1 kidney cancer, 1 gastric adenocarcinoma, 2 lung cancers)
Congenital agenesis of the pelvis	1 (10%)
Anticoagulation therapy	
Warfarin	2 (20%)
DOAC	4 (40%)
Antiplatelet therapy	0 (0%)

**Table 2:** Indications for chest drain placement

Indication	Number of patients (%)
Pleural empyema	3 (30%)
Hydropneumothorax	1 (10%)
Massive malignant pleural effusions	6 (60%)

### Procedural technique

**Fig. 1:**
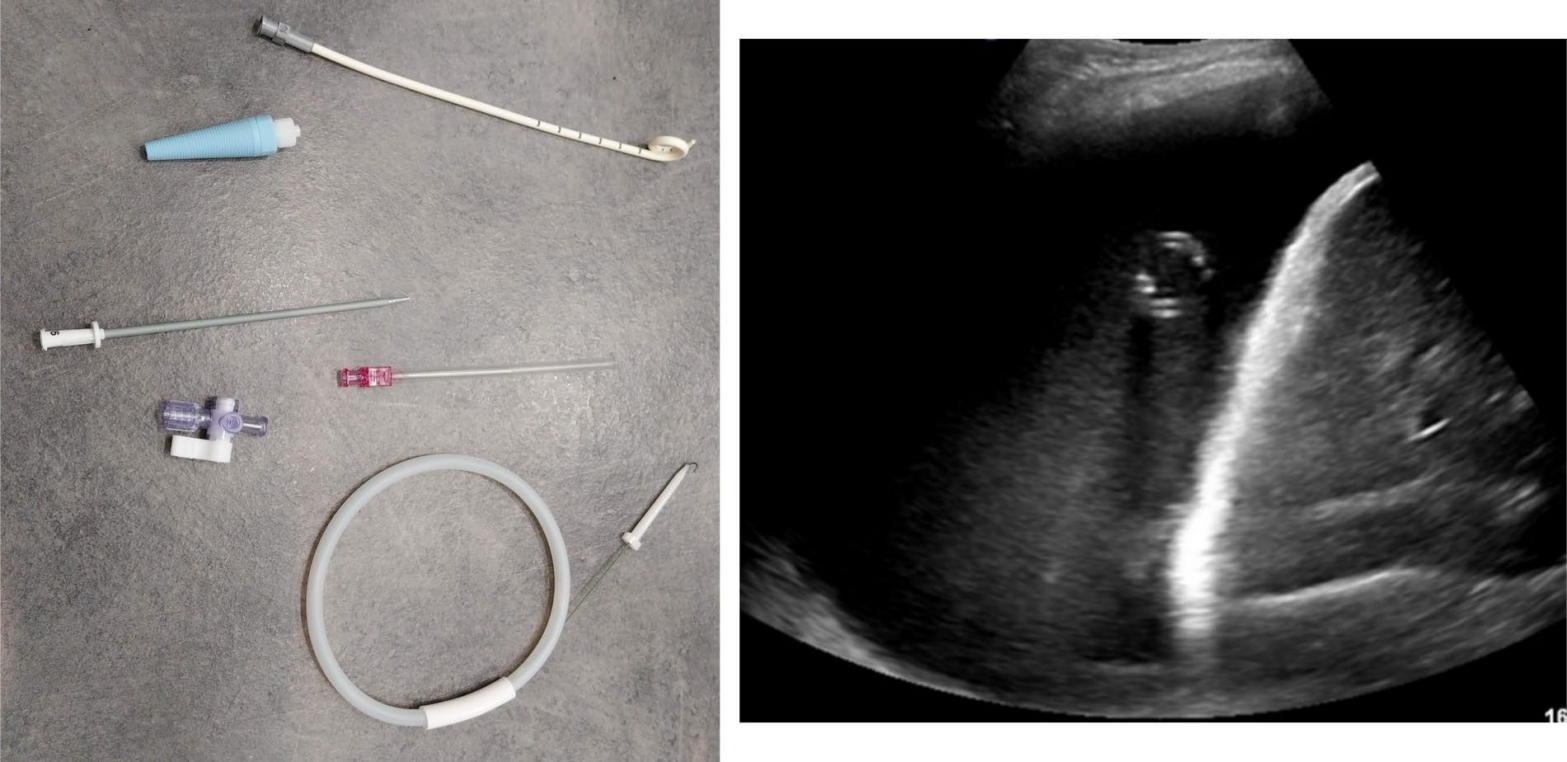
Small bore chest drain placement technique with ultrasound. Patients were positioned appropriately, and a detailed ultrasound examination was conducted to identify the optimal insertion site. Local anesthetic was administered at the chosen site. Under ultrasound guidance, a needle was advanced into the pleural space and a guidewire was inserted through the needle. Then, a dilator was used over the guidewire to prepare the tract. The small-bore chest drain was advanced over the guidewire into the pleural space. Correct placement was confirmed by ultrasound imaging and, where indicated, chest radiography

All procedures were performed using the Seldinger technique. In 4 cases, the chest drain was placed entirely under ultrasound guidance, while in 6 cases, ultrasound was used to assist with the procedure. A 10.2 French drain was used in each instance. The procedure is described in Fig. 1.

### Procedural success and early complications

In all ten cases, the chest drain was successfully positioned using the Seldinger technique with the aid of ultrasound. No immediate complications were reported during the procedure. Specifically, there were no cases of intercostal artery injury, no injuries to abdominal organs. Iatrogenic pneumothorax due to accidental lung puncture was avoided. No subcutaneous hematomas were observed.

### Post-procedural complications and management

At 24 h post-procedure, the following complications were noted (Table 3): two patients developed a mild hydro-pneumothorax attributed to rapid re-expansion of the lung. After thoracic surgeon consultation from the HUB hospital, those cases were managed conservatively with careful follow-up, and no invasive intervention was required. Accidental displacement of the drain was observed in two cases; however, these events did not result in any significant clinical deterioration, and the drains were repositioned or replaced as necessary. Thoracoscopy was considered in six cases. However, it was ultimately performed in only two patients. In the remaining four cases, further diagnostic evaluation in the internal medicine department was deemed sufficient.

**Table 3:** Post-procedure complications at 24 h

Complication	Number of patients	Management/outcome
Hydro-pneumothorax	2	Managed conservatively with careful follow-up; no invasive intervention required
Drain displacement	2	Drains were repositioned or replaced as necessary; no significant clinical deterioration observed
Thoracoscopy considered	2	

### Patient disposition and clinical implications

None of the patients died during their hospital stay. Four patients were discharged home; two patients were transferred to the infectious diseases ward; two patients were transferred to the thoracic surgery ward for further management. Two patients were transferred to hospice care due to advanced oncological disease (Table 4).

**Table 4:** Patient disposition and clinical implications

Disposition	Number of patients (%)
Discharged	4 (40%)
Transferred to the infectious diseases ward	2 (20%)
Transferred to the thoracic surgery ward	2 (20%)
Transferred to hospice	2 (20)

## Discussion

Our retrospective analysis indicates that the placement of small-bore chest drains using the Seldinger technique under ultrasound guidance is both feasible and safe when performed by internal medicine physicians with appropriate interventional ultrasound expertise. In our series, all ten procedures were successful, and there were no immediate life-threatening complications. The key advantage of using ultrasound lies in its ability to provide real-time visualization, reducing the risk of accidental puncture of the lung or injury to adjacent structures. This is particularly relevant in patients with complex pleural effusions, where anatomical distortions can increase procedural difficulty.

Although immediate complications were absent in our series, we observed a few delayed complications. In 2 patients, a mild hydro-pneumothorax developed within 24 h due to rapid re-expansion of a chronically collapsed lung. This phenomenon is well-documented in the literature, particularly in cases where a large volume of pleural fluid is removed rapidly from a lung with long-standing atelectasis. In our study, these cases were managed conservatively with close observation and follow-up imaging. Recent ERS/ESTS statement reported that there is sufficient evidence that 12–14 Fr chest tubes are efficient as a first-line intervention in pleural infection [11]; however, chest drains < 12 Fr are usually avoided to minimize risk of blockage and dislodgement [11]. In our sample, by using 10.2 Fr drains, there was no case of blockage, but 2 cases of accidental displacement. Although these displacements did not lead to significant clinical consequences, they highlight an area for procedural improvement. These findings are consistent with previous studies that have reported low complication rates with ultrasound-guided chest drain placement when performed by experienced operators.

The ability of internal medicine physicians to perform ultrasound-guided chest drain placement has significant clinical and logistical implications. In our cohort, 80% of the patients were managed entirely within the internal medicine department or related wards such as infectious diseases and hospice care. This capability reduces the need for hospitalization in or transfer to thoracic surgery wards, which can be particularly beneficial in resource-limited settings. For patients with advanced oncological diagnoses—some of whom received their diagnosis for the first time during hospitalization—this approach facilitates prompt palliative and supportive care, thereby improving overall patient comfort and reducing hospital stay duration.

Our experience underscores the importance of specialized training in interventional ultrasound for internists. As procedural responsibilities expand beyond traditional boundaries, it is imperative that internal medicine physicians acquire the necessary skills to perform these interventions safely. Formal training programs, simulation-based learning, and supervised clinical practice are essential to build competency and ensure patient safety.

Traditional management of complex pleural effusions has often relied on large-bore chest tubes inserted by surgical or interventional radiology teams. While effective, these procedures are more invasive, associated with higher patient discomfort, and carry a greater risk of complications such as bleeding and injury to adjacent structures [12]. In contrast, our study demonstrates that small-bore chest drains, when inserted under ultrasound guidance, offer a less invasive alternative with an excellent safety profile. This approach is particularly advantageous in the internal medicine setting, where prompt management is crucial, and transfer to specialized departments may delay care.

## Literature review

By analyzing original works published in the last 10 years (Table 5), failure rates of small bore chest tube (SBCT) are generally comparable to large bore chest tube (LBCT) in many settings, with some studies indicating slightly higher reintervention rates for SBCT in trauma [13, 14]. However, in non-traumatic pleural collections, SBCT appears to perform favorably with high success rates [13, 14]. While both SBCT and LBCT have distinct complication profiles, overall complication rates appear similar [5, 15, 16]. SBCT may reduce the risk of certain insertion injuries but may carry a higher risk of malposition or pneumonia [5, 15, 16]. SBCT generally results in shorter drainage durations and may contribute to a reduction in hospital length of stay, particularly in surgical settings [17, 18]. Patients receiving SBCT tend to report lower pain and better overall experience scores compared to those with LBCT [9, 14]. Both techniques are effective; the Seldinger method may provide a slight benefit in patient comfort and earlier drain removal, although differences are not robust across all endpoints [9]. Lewis et al. [19] demonstrated that in children with empyema, ultrasound-guided pigtail catheters (SBCT) achieved high success rates (93–98.2%) with minimal complications. Moreover, Hamad & Alfeky [13] showed excellent drainage success (95.98%) with SBCT in malignant and parapneumonic effusions, supporting their use in a non-trauma setting.

**Table 5:** This summary table captures the key methodological approaches, populations, interventions or comparisons, and primary outcomes across the different studies assessing chest tube and pleural catheter selection and outcomes in the last 10 years

Study (author, year)	Study design and methods	Population	Intervention/comparison	Key outcomes/results
Almusally et al. (2024) [5]	Cross-sectional electronic survey	Thoracic surgeons (49.1%) and pulmonologists (50.9%) from tertiary-level institutions (82.1%)	Evaluation of chest tube size selection preferences for pleural effusion	Preference for SBCT (< 14 Fr) in 54.8%; drawbacks of SBCT: kinking (60%), blockage (70%); LBCT drawbacks: pain (64%); ultrasound guidance positively influenced SBCT selection (55%); LBCT complications: visceral/vascular injuries (55.7%), wound infection (45.3%), re-expansion pulmonary edema (43.3%), subcutaneous emphysema (57.5%); SBCT malposition (49.1%)
Messa et al. (2024) [8]	Retrospective chart review (2016–2021) at a Level 1 Trauma Center	341 patients; 87.1% received LB tubes	Comparison between SBCT and LBCT chest tubes for traumatic PTX, HTX, or HPTX	LB tubes more often used in penetrating injuries with higher ISS and thoracic AIS; LB tubes had a higher incidence of retained hemothorax; no significant differences in tube failure or insertion-related complications
Congedo et al. (2023) [9]	Prospective, non-randomized, non-inferiority study	117 patients (72 in Seldinger group, 45 in Trocar group)	Comparison of Seldinger vs. Trocar technique for small-bore pleural drains	Similar procedure times (7.93 vs. 7.09 min, *p* = 0.33) and pain (VAS ~ 2.2 vs. 2.8, *p* = 0.07); day 2 pain higher in one group (*p* = 0.04); no differences in complications or residual effusion; drain removal rates tended to be lower in Seldinger group (11.6% vs. 25%, *p* = 0.063); overall removal time ~ 8.87 days
Wu et al. (2022) [17]	Prospective observational study	131 patients undergoing surgical stabilization of rib fractures with VATS	Comparison between 32-Fr chest tubes (Group 1, *n* = 65) and 14-Fr pigtail catheters (Group 2, *n* = 66) for postoperative drainage	Group 2 (small-bore) had significantly shorter drainage duration (3.11 ± 1.31 vs. 5.08 ± 2.47 days, *p* = 0.001) and shorter hospital length of stay (8.18 ± 2.44 vs. 10.38 ± 2.90 days, *p* = 0.001)
Kulvatunyou et al. (2021) [14]	Multicenter randomized clinical trial	119 patients with traumatic hemothorax (56 randomized to 14-Fr percutaneous catheters vs. 63 to 28–32 Fr chest tubes)	Comparison of small (14 Fr) percutaneous catheter vs. large (28–32 Fr) open chest tube	Failure rates similar (11% vs. 13%, *p* = 0.74); secondary outcomes similar except for patient experience: percutaneous catheters had lower insertion perception scores (median 1 vs. median 3, *p* < 0.001)
Hamad and Alfeky (2021) [13]	Retrospective observational study	369 patients; 398 small-bore pleural catheters inserted for pleural collections	Use of small-bore catheters for pleural drainage	Drainage success in 95.98% of cases; 6 cases required decortication; catheter reinsertion for dislodgment in 0.50% and obstruction in 0.75%; 15.58% experienced chest pain; average drainage duration was 3.5 days
Maezawa et al. (2020) [15]	Retrospective observational study in the ED	102 patients (107 tube thoracostomies) for chest trauma	Use of small-bore tube thoracostomy (≤ 20 Fr) vs. traditional methods	Mean tube duration was 3.9 ± 1.8 days; tube-related complications occurred in 7.8% (retained HTX in 3.9%, unresolved PTX in 3.9%); no tube obstruction-related complications
Orlando et al. (2020) [16]	Retrospective multicenter cohort study	Patients with delayed hemothorax: 160 SB patients (191 tubes) vs. 60 LB patients (72 tubes)	Comparison of small-bore vs. large-bore chest tubes	Overall complication rates similar (SBCT: 18% vs. LBCT: 14%, *p* = 0.42); LBCT had higher risk of VATS, SBCT higher risk of pneumonia; least squares mean drainage rate lower in SBCT (52.2 vs. 213.4 mL/hr, *p* < 0.001) but median rates were similar; initial output volume similar
Yang et al. (2020) [18]	Prospective study	88 consecutive patients undergoing single-incision thoracoscopic lobectomy for lung cancer	Comparison of small-bore pigtail catheter (smaller drain group) vs. conventional 26-Fr chest tube (larger drain group)	Smaller pigtail group had shorter drainage time, higher drainage volume in first 2 days, lower incidence of pleural effusion after 2 weeks (4.54% vs. 25%, *p* = 0.007), and significantly lower pain score on day 3 (*p* < 0.001); no differences in air leak time or subcutaneous emphysema
Lewis et al. (2018) [19]	Retrospective review over 16 years at a single center	285 pediatric patients with parapneumonic effusion/empyema; 303 drains placed	Real-time ultrasound-guided pigtail catheter placement for complicated parapneumonic effusion/empyema	Single drain success in 93% of patients (98.2% success with 2–3 drains); 5 peri-insertion complications (none significant); 5 patients required surgical intervention; post-2012, all children successfully treated with single-tube drainage, with no surgical conversions

*SBCT* small-bore chest tube, *LBCT* large-bore chest tube, *HTX* hemothorax, *PTX* pneumothorax, *HPTX* hemopneumothorax

Therefore, across multiple settings—from trauma to postoperative and infectious/empyema cases—SBCT (or pigtail catheters) perform comparably to LBCT in terms of failure rates and overall complications. While LBCT may carry higher risks of insertion-related injuries (e.g., visceral/vascular injury, re-expansion pulmonary edema), SBCT are more likely to be malpositioned or associated with pneumonia. However, these differences tend not to affect overall clinical outcomes significantly. SBCT tend to result in shorter drainage durations and lower hospital lengths of stay, especially in surgical patients. Moreover, patients report better tolerability and lower pain scores, particularly when the Seldinger technique is used. Although both Seldinger and Trocar methods are viable, subtle advantages in patient experience and early drain removal appear to favor the Seldinger technique for SBCT placement.

## Limitations of the study

This retrospective analysis has several limitations. First, the sample size of ten patients is relatively small, limiting the generalizability of the findings. Second, the study is conducted at a single center with experienced operators, and outcomes may differ in other settings with less interventional ultrasound expertise. Finally, the lack of a control group receiving traditional chest tube placement precludes a direct comparison of complication rates and outcomes. Future prospective studies with larger cohorts and comparative designs are needed to validate these findings further.

## Conclusions

Our retrospective analysis indicates that ultrasound-guided small-bore chest drain placement performed by internal medicine physicians with interventional ultrasound expertise is a feasible and safe procedure in the management of massive and complex pleural effusions. The high success rate and low incidence of immediate complications, coupled with the minimal delayed complications observed, can support the adoption of this technique as a first-line intervention in hospital settings lacking specialized thoracic surgery services. The results of our study reinforce the concept that the use of ultrasound guidance not only improves the accuracy of drain placement but also minimizes the risk of complications such as iatrogenic pneumothorax and vascular injury. Although re-expansion hydro-pneumothorax and accidental drain displacement were noted as potential issues, these complications were manageable with conservative measures and did not result in significant clinical deterioration. To the best of our knowledge, this is the first study that has analyzed the use of small-bore chest drains in an internal medicine setting for pleural effusions of non-traumatic causes. Notably, the ability to perform this procedure within the internal medicine department allows for comprehensive diagnostic evaluation and therapeutic management without the need for transfer to specialized units. This is particularly beneficial for patients with complex oncological diagnoses or those requiring urgent palliative care. With appropriate training and adherence to standardized protocols, this procedure can significantly improve patient care by providing a minimally invasive, effective, and safe method for managing complex pleural effusions.

## Data Availability

Data are available upon request from authors.
